# Are We Adequately Testing Essential Oils as Insecticides in the Laboratory? Bridging the Gap Between Laboratory Bioassays and Field Applications

**DOI:** 10.3390/plants15010084

**Published:** 2025-12-26

**Authors:** Alejandro Lucia, Eduardo Guzmán, Ariel C. Toloza

**Affiliations:** 1 Instituto de Ecología y Desarrollo Sustentable (INEDES, CONICET-UNLu), Luján, Buenos Aires B6700, Argentina; 2 Laboratorio de Investigaciones en Madera (LIMAD), Facultad de Ciencias Agrarias y Forestales, UNLP, Buenos Aires B1900, Argentina; 3 Departamento de Química Física, Universidad Complutense de Madrid, 28040 Madrid, Spain; eguzmans@ucm.es; 4 Instituto Pluridisciplinar, Universidad Complutense de Madrid, 28040 Madrid, Spain; 5 Centro de Investigaciones de Plagas e Insecticidas (UNIDEF, CONICET), J. Bautista de La Salle 4397, Villa Martelli, Buenos Aires B1603ALO, Argentina; atoloza@conicet.gov.ar

**Keywords:** bioassays, biopesticides, essential oils, insecticides, real-world conditions, spraying

## Abstract

Essential oils (EOs) have been extensively studied as potential alternatives for insect pest management. In recent years, research on these natural compounds has increased substantially. However, despite numerous studies demonstrating the insecticidal properties of EOs under laboratory conditions, their practical application remains limited. This discrepancy highlights a significant gap between experimental findings and the development of commercially viable products. Several factors have been proposed as the basis for this gap, including the absence of positive controls to compare their effectiveness (i), the imperative need to develop new formulations (ii), and the potential toxicity of many to non-target organisms (iii). This work focuses on why the information obtained in the laboratory has not translated into the biopesticide market. A key issue is the difficulty of applying laboratory knowledge in adapting to field-like scenarios, such as spray quality (droplet size and volume), the nature of the application solvent used in the sprayer tank, and the way the insect is exposed to the insecticide (i.e., the type of laboratory bioassay selected). This challenge is primarily due to researchers’ limited understanding of the application techniques used in field settings to manage specific insect pests. Many laboratory bioassays designed to measure effectiveness do not accurately reflect field conditions; instead, they often create scenarios that artificially enhance effectiveness. This results in an unrealistically high effectiveness estimate of the true potential of EOs in controlling the targeted insects.

## 1. Introduction

Essential oils (EOs) have been studied for decades as potential alternatives for insect pest control [1,2,3]. The number of publications has increased significantly in recent years, particularly reviews on more specific topics, such as their historical development, current usage patterns and regulatory frameworks [4,5,6,7,8,9]; advantages over conventional pesticides, potential, constraints, and limitations [10,11]; modes of action [12,13,14,15]; formulation strategies and applications [16,17,18,19,20,21]; commercial opportunities [22,23]; and, finally, their effects on non-target organisms [24,25] and associated ecological costs [26]. Despite this extensive literature on the bioactivity of EOs as insecticides, only a few products have successfully reached the market. This discrepancy highlights a widely recognized gap in translating laboratory findings into practical applications [2,27,28].

Several reasons have been proposed to explain this gap, including: (i) the lack of standardized positive controls, i.e., reference treatments using well-characterized active ingredients with pesticidal activity applied with the same technique and dosage framework as the tested molecules, allowing for meaningful performance benchmarking; (ii) the urgent need to develop improved and stable formulations, and (iii) the potential toxicity of some EOs toward non-target organisms [24,25], despite their favorable environmental profile [27,28,29,30]. Additional challenges include variability in EO composition due to plant chemotypes, extraction methods, and storage conditions, which can greatly influence insecticidal efficacy [11,28]. Moreover, limited knowledge of formulation stability, solubility, and delivery methods may further hinder the translation from laboratory to field conditions.

This work aims to elucidate why information obtained under laboratory conditions is not successfully translated into biopesticides suitable for professional use. The application of laboratory findings to real-world scenarios is often hindered by several factors, including a lack of expertise in appropriate pest management techniques, inadequate formulation methods, and methodological flaws in experimental design. Common conceptual errors include the absence of positive controls and expressing doses in units that are practical only under laboratory conditions.

Many laboratory bioassays used to evaluate EO efficacy do not accurately simulate field-realistic conditions, i.e., experimental conditions that replicate key operational parameters of field applications, including application technology, formulation, dosage expression, and exposure route, rather than merely environmental settings. As a result, experiments often overestimate efficacy, introducing a methodological bias that has been highlighted in previous reviews [7,31]. This is largely due to the high vapor pressure of EOs, which produces strong fumigant activity when insects are exposed via inhalation. At the high doses used in laboratory tests, EOs are also likely to elicit repellency [32,33]. While these conditions ensure measurable effects in the laboratory, they often do not provide meaningful insights into real-world performance. Furthermore, interactions with environmental factors such as temperature, humidity, and UV light, which can drastically influence EO activity, are rarely considered in laboratory settings.

In this context, the present review critically evaluates whether commonly used laboratory bioassays adequately reflect real-world application scenarios, identifies key methodological gaps, and proposes practical recommendations to enhance the ecological and operational relevance of future studies. By bridging the gap between laboratory assessment and field applications, this work aims to provide a clear roadmap for the development and adoption of essential oil (EO)-based insecticides.

To achieve these objectives, we conducted a structured bibliometric literature search designed to capture the methodological diversity of plant-based insecticidal studies, with particular emphasis on EOs. The search covered the period from 2005 to 2025 to ensure the inclusion of contemporary methodological trends and debates, while also capturing foundational reviews and key methodological studies from the preceding decades. The primary focus of the review was to compare the main laboratory bioassays used to evaluate insecticidal activity, namely contact bioassays via topical application (direct contact), contact bioassays using impregnated surfaces (indirect contact), and fumigant bioassays, and to critically relate these approaches to application methods used under realistic or field conditions. We also aimed to identify gaps in experimental design and data report that limit the predictive value of laboratory results for practical pest management.

Literature searches were performed in the Scopus, Web of Science, and Google Scholar databases using combinations of relevant keywords, including “plants,” “plant extracts,” “essential oils,” “botanical insecticides,” “insecticidal activity,” “contact bioassay,” “topical application,” “impregnated surfaces,” “fumigant bioassay,” “spraying,” and “field application.” Studies were selected based on their relevance to the methodological aspects under discussion and their ability to illustrate specific experimental designs, exposure routes, and application techniques. Rather than applying strict exclusion criteria, we adopted an inclusive approach, incorporating a sufficient number of representative publications to adequately cover each methodological framework and plant-based system addressed. This strategy ensured that all selected references contributed meaningfully to the comparative analysis and to a broader understanding of how laboratory bioassays for EO-derived insecticides relate to practical field applications. Overall, this review aims to provide a practical guide for researchers designing laboratory with improved predictive power for field performance, thereby supporting the development of viable EO-based pest management solutions.

## 2. Application of Insecticides Under Real-World Conditions

Incorrect use of insecticide application techniques under controlled laboratory conditions may lead to an overestimation of EO performance, as such outcomes rarely reflect operational field conditions. Accurate evaluation of EO efficacy against pest insects requires a clear understanding of the laboratory application method used and the extent to which it represents recommended field practices.

Insecticide formulations or commercial products can be applied using different techniques, each of which would ultimately influence the distribution required to achieve lethal or sublethal effects on insect pests. There are three main techniques for applying formulated insecticides: dusting, spraying, and fumigation [34,35]. Dusting uses a powder formulation, spraying distributes a liquid in fine droplets, and fumigation disperses a gas or smoke. Common fumigants include phosphine, methyl bromide, and fumigant pots. Each technique results in distinct exposure patterns, which must be considered when extrapolating laboratory bioassay results to field applications.

Spraying is the most commonly used application technique, which involves the atomization of a liquid into droplets that are dispersed into the environment [36,37]. Due to its widespread use and significant impact on insecticidal effectiveness, a thorough understanding of the spraying process is crucial for assessing the efficacy of EOs and extrapolating laboratory findings to field applications.

### 2.1. Spraying Technology

In spraying, droplet size is determined by internal and external pressure, nozzle design, and the type of energy used for atomization. Droplet characteristics influence coverage and deposition, and ultimately determine insecticidal effect on target pests. Spraying can be divided into two main subgroups based on droplet size and the volume of mixture applied.

#### 2.1.1. High-Volume Spraying (HV)

High-Volume (HV) spraying is a technology that applies more than 400 L/ha [34], typically producing droplet sizes in the range of 200–500 μm. The droplet size is determined by the application volume and nozzle type [38]. This method, also known as conventional or residual spraying, is often conducted with manual applicators. Its primary objective is to thoroughly wet the treated surface to maximize contact with target insects.

#### 2.1.2. Ultra-Low-Volume Spraying (ULV)

Ultra-low-volume (ULV) spraying is characterized by application volumes of less than 5 L/ha. This spraying method produces droplets with diameters ranging from 0.1 to 50 μm [39]. The aim is to create very small droplets that stay suspended in the air for long periods, increasing the chances of contacting flying insects. ULV spraying is often used outdoors with hand-held, vehicle-mounted, or aircraft-mounted equipment. It is also commonly used indoors with hand-held devices and aerosol formulations.

Space spraying is a related technique that disperses liquid insecticide into the air as fine droplets, each less than 50 μm in diameter [40]. This method is only effective while the droplets remain airborne. As a result, droplet sizes are kept small, typically between 0.1 and 50 μm in diameter, with about 80% ranging from 0.1 to 30 μm [41]. According to the US Environmental Protection Agency, ULV also refers to a total spray volume of 1.9 L/ha or less.

The effectiveness of space spraying drops significantly when the droplet size is below 5 μm or above 25 μm (volume median diameter, VMD). For mosquito control using ground equipment, the optimal droplet size to achieve at least 90% mortality is 10–15 μm VMD. For aerial applications, slightly larger droplets (5–25 μm VMD) are more effective [42].

The operational parameters, target scenarios, and implications for laboratory simulation of the primary application techniques discussed are synthesized in Table 1. This comparison highlights the importance the necessity of selecting a laboratory bioassay that aligns with the specific physical and operational constraints of the intended field application method.

### 2.2. Selection of the Spraying Method Based on the Target Insect

Defining the optimal droplet size for field application is challenging due to the wide variety of target pests and habitats. Recommended droplet sizes vary according to the target organism: flying insects typically require droplets of 1–50 μm, insects found on foliage need 30–50 μm, and applications to foliage or soil, where minimizing drift is important, typically requires larger droplets in the range of 250–500 μm [34].

The uptake of EOs occurs via direct or indirect contact. In direct contact, insects are impacted by spray droplets, whereas in indirect contact, they acquire the insecticide by walking or resting on treated surfaces. In both cases, insecticide penetration occurs through the cuticle and is transported to the central nervous system, where the active compounds exert their toxic effects. The efficiency of EO uptake is influenced by insect morphology and behavior, and the physicochemical properties of the EO formulation.

Contact insecticides are generally most effective against soft-bodied insects such as lepidopteran larvae but show reduced efficacy against species with thick or heavily sclerotized exoskeletons, or those that produce protective waxy layers (e.g., scale insects) or foams (e.g., spittlebugs). Because contact insecticides are largely confined to the leaf surface, they primarily affect existing pest populations through cuticular uptake, but provide limited protection against new infestations. These insecticides are generally highly lipophilic and minimally systemic, meaning that new plant growth is typically unprotected after treatment [43].

## 3. Application of Insecticides Under Laboratory Conditions

This review examines not only studies that evaluated EOs as alternatives to conventional insecticides, but also research aimed at expanding fundamental scientific knowledge rather than generating directly applicable results. The latter studies provide valuable insights into experimental variables that are often overlooked in laboratory bioassays and that critically limit the extrapolation of laboratory findings to field-like scenarios.

The discussion above underscores the complexity of optimizing application techniques for effective pest control under field conditions. While field trials require precise calibration and adherence to established protocols, laboratory studies often lack similar methodological rigor. Achieving meaningful alignment between laboratory assays and field performance demands experimental conditions that realistically reflect real-world paths, environmental parameters, and application methods.

EOs and/or their individual components can enter the insect body through three main routes: inhalation via the respiratory system [44]; contact with treated surfaces, where toxicity occurs as insects move across or rest on contaminated areas [45]; or direct topical application onto the insect cuticle [46]. Understanding which exposure route predominates in the field is critical for designing laboratory bioassays that produce transferable results.

The following sections provide a detailed description of laboratory bioassays used to test toxicity through these different routes of insecticide penetration, emphasizing their methodological constraints and the challenges in extrapolating laboratory findings into effective field applications.

### 3.1. Contact Bioassays via Topical Administration (Direct Contact)

In laboratory bioassays assessing EO toxicity via topical administration, the objective is to replicate the spraying technique used under practical-use conditions. In the field, spraying requires strict control of parameters, such as flow rate, droplet size, humidity, and temperature to ensure effective pest coverage. However, laboratory topical applications using droplet-based methods (e.g., syringe, sprayers, or Potter towers) only approximate real scenarios.

It is important to note that, although laboratory methods can be highly precise, especially when validated techniques and equipment that control variables like volume, pressure, and dosage (expressed as mg a.i/cm^2^ or cm^3^) are used [47,48,49], they often fail to replicate realistic field conditions. The most commonly used equipment for simulating field spraying includes the Potter spray tower (Burkard, Uxbridge, UK) for high-volume spraying [50], and the Peet–Grady chamber for ultra-low volume (ULV) aerosol applications [51].

**Figure 1:**
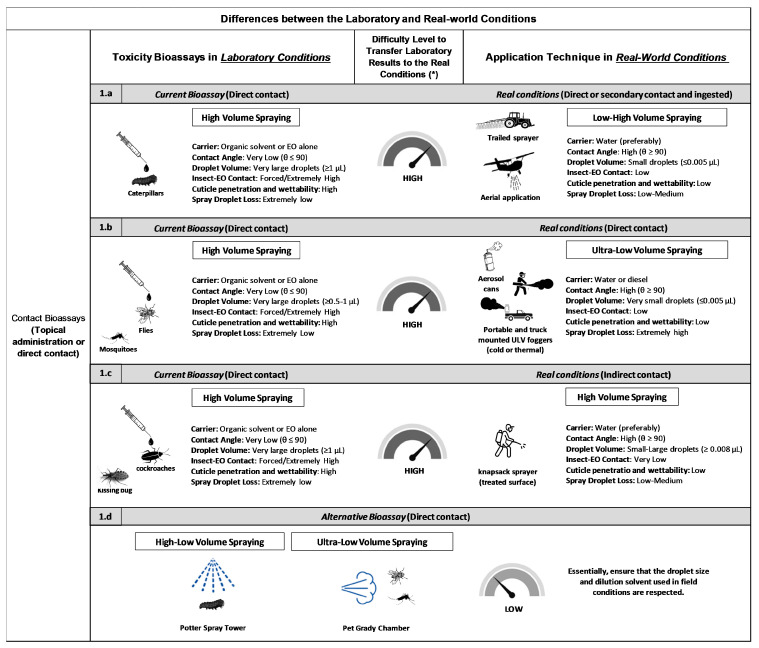
(**a**–**d**) Clear differences are observed between laboratory conditions used to test the insecticidal activity of essential oils against pests (via contact bioassays such as topical application or direct contact) and the techniques recommended for real-world application. (*) Difficulty Level to Transfer Laboratory Results to the Real Conditions: This refers to how well laboratory toxicity bioassays replicate field application techniques. A low difficulty indicates the lab bioassay closely matches the real-world application, and the data is more applicable. Carrier: In pesticides applied as sprays, the carrier is the diluent in the sprayer tank; in labs, it is typically the dilution solvent. Contact Angle: A contact angle less than 90° implies good wetting, while an angle greater than 90° signals poor wetting. Droplet Volume: In laboratory conditions, the exact dose is delivered with a micro-applicator or micro-syringe. In the field, it refers to droplet volume managed by applying the correct spraying technique for the target insect. Insect-EOs Contact: This parameter simulates how much of the active ingredient reaches its biological target (the insect cuticle), integrating factors like carrier type, droplet size/volume, and loss from evaporation between the nozzle and target site. Cuticle Penetration and Wettability: Refers to how the droplet spreads and penetrates upon reaching the cuticle of the insect. Spray Droplet Loss: Represents spray droplet loss from drift, evaporation, miss, shatter, bounce, and runoff.

In real spraying operations, the application quality is determined by three main parameters: (i) the nature of the application solvent in the sprayer tank (oil- or water-based), (ii) the flow rate, and (iii) droplet size and volume. Certain environmental factors, such as wind speed, temperature, and relative humidity, can influence drift and evaporation, directly affecting application efficacy [37]. These considerations are often overlooked in laboratory assays. Typically, spraying aims to affect insects either directly (through contact during spraying) or indirectly (via contact with treated surfaces). Figure 1a highlights the primary differences between laboratory and field spraying parameters, including solvent type, flow rate, and droplet characteristics.

A second major discrepancy, in which laboratory techniques fail to reflect operational conditions, involves the treatment of flying insects (e.g., mosquitoes and flies) using hand-operated micro-applicators. Due to differences in droplet size and volume, these laboratory methods do not accurately represent the ULV spraying techniques recommended for mosquito or fly control in the field (Figure 1b). Spatial spraying methods, such as ULV generate aerosolized droplets between 5 and 50 μm, which stay airborne long enough to contact flying insects without leaving residues (e.g., spraying mosquitoes or using canister sprays for flies). However, many laboratory studies continue to apply large droplets (≥0.5–1 μL) topically using hand micro-applicators [52,53,54,55,56]. In comparison, ULV field operations expose insects to much smaller droplets (≤0.005 µL). The higher droplet volume used in laboratory assays, along with the use of organic solvents that enhance EO penetration, results in an overestimation of EO toxicity under laboratory conditions.

A third point of discrepancy involves walking insects, such as cockroaches and triatomines, which are frequently subjected to topical application in laboratory tests even though, in the field, they are primarily exposed to insecticides indirectly by contacting treated surfaces (see Figure 1c). This methodological gap affects species like *Blattella germanica* (Linnaeus) [57,58,59], *Triatoma infestans* (Linnaeus) [60,61], and *Rhodnius* sp. (Stål) [62].

Bridging the gap between laboratory and field conditions requires adapting laboratory setups to better reproduce field spray parameters. For the scenarios illustrated in Figure 1a,b, researchers should use formulated EO products diluted in water and applied with standardized spray systems (e.g., Potter spray tower or Peet–Grady chamber), to reproduce droplet sizes and application volumes comparable to those used in the field. This adjustment would result in more accurate laboratory data that can be reliably extrapolated to field performance (Figure 1d). For the scenario depicted in Figure 1c, the direct topical application of undiluted or solvent-based EOs onto the insect cuticle is not a realistic approach for toxicity evaluation, as many species are exposed to insecticides only by walking across treated surfaces rather than through direct spraying.

### 3.2. Contact Bioassays via Impregnated Surface (Indirect Contact)

Laboratory bioassays used to evaluate the indirect contact toxicity of conventional insecticides typically involve impregnating a surface (such as filter paper or glass) with the test product. The treated surface is placed in a container, into which insects are introduced and allowed to walk for a specified period [63]. Following exposure, insects may be transferred to untreated systems with water and food, and monitored for intoxication symptoms (hyperactivity, incoordination, tremors, lethargy, knockdown, and death). However, several limitations arise when using this technique to assess the indirect contact toxicity of EOs.

**Figure 2:**
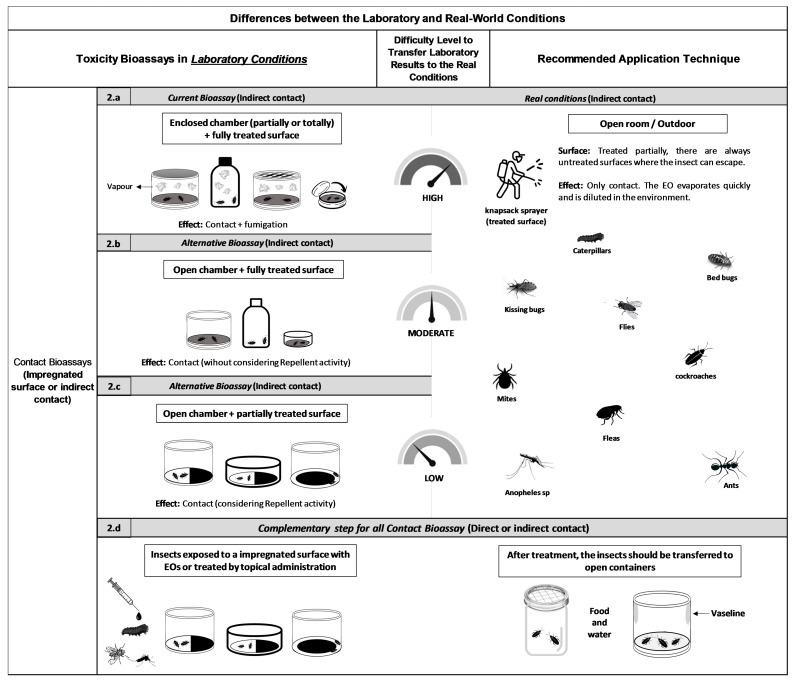
(**a**–**d**) Differences between the laboratory conditions used to test the insecticidal activity of essential oils against various pests using contact bioassays (such as impregnated surfaces or indirect contact methods) and the application techniques recommended for applied contexts.

To accurately determine the toxic effects resulting from indirect contact, it is essential to exclude the fumigant action of EOs, as this can be affected by several factors: (i) the size of the container housing the insects; (ii) the type of treated surface treated; (iii) the number of insects placed in the container; (iv) the material used to seal the container; and (v) the duration of insect exposure to the treated surface. When a large number of insects are confined for extended periods in a small, partially or fully sealed container, vapor accumulation increases, making inhalation toxicity significant. Consequently, container geometry-notably its size and shape-affects both insect movement and vapor concentration, thereby impacting total mortality. As a result, mortality observed in surface contact assays may often be caused more by inhalation of EO vapors than to contact toxicity. In contrast, container type is less critical when testing conventional insecticides, as these compounds generally act within seconds or minutes, allowing limited time for vapor effects. In these assays, treated surfaces such as filter paper are commonly placed in partially or fully sealed chambers after solvent evaporation. These systems included small glass vials [45], Petri dishes [64,65,66,67], Petri dishes sealed with Parafilm [68,69], perforated Petri dishes [70], bottles [71], and glass jars [72], among others. When insects are exposed to EO-treated surfaces within a closed containers they are also exposed to EO vapors. This often results in contact toxicity at high concentrations and can also cause repellency [32,33,73,74,75] and fumigant activity [76,77,78] (Figure 2a). These EO properties can deter insects from treated areas under field conditions. Consequently, in natural environments, insects are unlikely to directly contact or inhale EOs, as they rarely occupy confined spaces. Therefore, both repellent and fumigant effects significantly confound laboratory assessments of true contact toxicity. Additionally, testing insects in an enclosed chamber prevents distinguishing between toxicity due to cuticular absorption and that resulting from the inhalation through the spiracles. For example, the CDC bottle bioassay [79,80] commonly used to assess EO efficacy against mosquitoes [71,81] was initially developed to determine discriminating doses in insecticide resistance assays. In this method, insects are confined in small glass bottles (approximately 250 cm^3^) coated with the test substance, which forces repeated contact with treated surfaces while simultaneously exposing them to vapors. The confined environment limits avoidance behaviors, often leading to an unrealistically high toxicity estimate. While contact bioassays are suitable for conventional insecticides that are non-repellent and lack fumigant activity, they are inappropriate for EOs, whose volatility and repellency compromise the assumptions these assays are based on.

Most recipients used to transfer insects after treatment with EOs are partially or fully closed. However, many studies do not transfer insects after the exposition to EOs, leaving them in the original closed or semi-closed arenas. This practice makes it difficult to distinguish between contact and inhalation effects [45,65,68,72,82]. In contrast, insects in natural environments usually inhabit open or ventilated spaces and can avoid treated areas. Therefore, closed-container tests do not accurately simulate real-world conditions. To better approximate field conditions, experiments should be conducted in open or ventilated chambers to minimize fumigant toxicity (Figure 2b), thus reducing the effect of inhalation. Additionally, providing untreated areas within the testing area allows insects to exhibit avoidance behavior, enabling a more accurate assessment of contact toxicity (Figure 2c).

A final consideration applies to both topical and surface-contact bioassays. Insects treated directly with EOs or exposed to EO-treated surfaces may retain enough volatile residue to saturate the atmosphere of the transfer container, unintentionally generating a fumigant effect. Only a few studies have implemented adequate ventilation to prevent this, using recipients with mesh or perforated lids [57,76], jars covered with metal grids, perforated plastic caps sealed with organza [83], mesh-covered cups [76], plastic boxes with perforated caps [84], gauze-covered boxes [52], or open Petri dishes [65], which help to reduce the confounding effects of vapor toxicity. For example, Araujo et al. [65] found that insect mortality with Piper oil in open Petri dishes was significantly lower than in closed Petri dishes, highlighting the impact of fumigant activity. Therefore, laboratory assays should replicate the droplet size and solvent dilution used in field conditions to produce realistic and transferable data (Figure 2d).

### 3.3. Fumigant Bioassay

Essential oil (EO) components exhibit moderate vapor pressures, allowing for partial volatilization and potential fumigant activity. Examples include α-terpineol (0.09 mmHg), γ-terpinene (0.93 mmHg), 4-terpineol (0.13 mmHg), p-cymene (1.35 mmHg), α-pinene (3.66 mmHg), and 1,8-cineole (1.34 mmHg) [78]. Due to their volatility, EOs can quickly enter the insect body through inhalation, producing fumigant-like effects across various insect species. This property enables the assessment of EO effects on a wide range of pest insects, regardless of their behavior or feeding habits [85,86,87]. However, to obtain meaningful results from laboratory fumigation bioassays, it is important to first determine if the target insect species is actually controlled by fumigant products in field-like scenarios. True fumigation requires prolonged exposure, often for days or weeks, in a fully sealed environment, conditions rarely found outside storage systems. Consequently, only a limited number of insect pests are effectively controlled by fumigants in practice, mainly those associated with stored cereals, such as *Tribolium castaneum* (Herbst), *Rhyzopertha dominica* (Fabricius), *Plodia interpunctella* (Hübner), *Sitophilus oryzae* (Linnaeus), and *Sitotroga cerealella* (Olivier). Fumigation remains a vital component of stored-grain protection programs, and traditionally relies on methyl bromide (MBr) and aluminum phosphide (AlP) [88].

**Figure 3:**
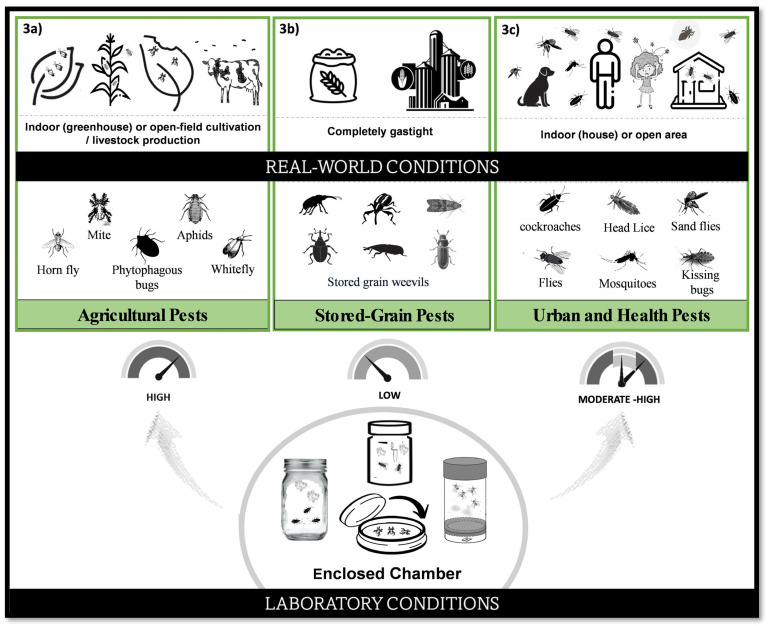
Differences between the laboratory conditions used to test the fumigant activity of essential oils against various pests and the application techniques recommended for real-world use. (**a**) Agricultural pests; (**b**) Stored-grain pests and; (**c**) Urban and health pests.

The fumigant activity of EO vapors has been widely tested on stored-grain pests using closed chambers (Figure 3b) [89,90,91,92]. However, plant-derived fumigants often lack the high vapor pressures required for efficient diffusion and penetration into infested commodities. For example, the vapor pressure of 1,8-cineole is below 1 mmHg at 20 °C, whereas synthetic fumigants such as phosphine (31,920 mmHg at 23 °C), methyl bromide (1250 mmHg at 20 °C), and sulfuryl fluoride (12,087 mmHg at 20 °C) exhibit much greater volatility and consequently higher efficacy [88].

Although closed chambers are appropriate for testing fumigant activity against stored-grain pests, their reduced volume prevents the use of standard positive control doses (e.g., MBr or AlP). Commercial formulations cannot be reliably scaled down for these miniature test chambers, which limits direct comparison between EO-based and conventional fumigants.

It is important to note that the presence of an insect in indoor environments (such as in a greenhouse or household) does not necessarily warrant evaluation under fully enclosed laboratory conditions. Such settings fail to accurately represent the semi-open environments typical of most indoor spaces. Nevertheless, many laboratory studies have tested agricultural or urban pests in fully enclosed conditions that poorly reflect their natural ecology (see Figure 3). Examples include *Nezara viridula* (Linnaeus) [93], *Tetranychus urticae* (Koch)*, Rhopalosiphum maidis* (Fitch)*, Bemisia tabaci* (Gennadius) [94], *Haematobia irritans irritans* (Linnaeus) [95], *Aedes aegypti* (Linnaeus) [96], *Musca domestica* (Linnaeus) [52], *Pediculus humanus capitis* (De Geer) [97], *Rhodnius prolixus* (Stål) [77], *Phlebotomus* sp. (Rondani y Berté) [98], and *Blattella germanica* (Linnaeus) [99]. An exception applies to mosquito control, where enclosed systems should be used exclusively to evaluate vapor-phase toxicity, as these products are specifically designed for volatile delivery methods such as mosquito coils, vaporizing mats, ambient emanators, and liquid vaporizers.

In fumigation bioassays, it must be acknowledged that insects will not experience such fully enclosed conditions in field settings. Therefore, using open or partially ventilated chambers in laboratory tests is more appropriate to avoid overestimating the efficacy of EOs. Closed-system bioassays artificially increase insect contact to EO vapors, leading to uptake primarily through inhalation, a pathway that is often irrelevant in practical-use conditions. As all EOs are volatile and exhibit some degree of fumigant activity, several precautions should be considered: (i) fumigant toxicity should not be evaluated in species that are not managed by fumigation in the field, as appropriate positive controls are unavailable; (ii) closed-container assays should be restricted to stored-grain pests, which regularly experience gastight conditions; (iii) EO toxicity should be assessed using devices that mimic real fumigant applications (such as coils or vaporizers), rather than spraying; and (iv) when contact and inhalation routes occur simultaneously, results cannot be extrapolated to field conditions, as each pathway requires independent dosage assessments.

In summary, although EOs exhibit volatility and fumigant effects under laboratory conditions, their real-world use is limited by low vapor pressure, rapid dissipation, and inconsistent formulation. Future research should focus on developing formulation technologies, such as microencapsulation, nanoemulsions, or slow-release matrices, to enhance vapor stability, diffusion, and persistence in semi-field and field conditions.

## 4. Suggestions for Testing EOs as Insecticides Under Laboratory Conditions

Effective insect pest control in the field relies on specific application techniques. To ensure that laboratory results are transferable to field conditions, these operational parameters must be accurately replicated. The following guidelines highlight key considerations for optimizing laboratory bioassays when evaluating EOs as insecticides:

1. **Replicate the field application technique in the laboratory.** The primary mode of insect control in the field, whether through direct contact during spraying, indirect contact with treated surfaces, or fumigation, should guide the design of the laboratory assay. Understanding the ecological context, such as insect behavior, feeding habits, and resting sites, is essential for realistic laboratory simulations.
  Briefly, the appropriate bioassay type should be selected based on the field control method.
  i.   Contact bioassays via topical administration (direct contact).
  ii.  Contact bioassays via impregnated surfaces (indirect contact).
  iii. Fumigant bioassays
  Appropriate bioassay selection ensures that exposure routes, dose delivery, and assessment endpoints closely match field-relevant scenarios.
2. **Specific considerations according to bioassay type**
  - Contact Bioassays via Topical Administration (Direct Contact)
    - Key variables related to spray application should be carefully controlled, including (i) the nature of the solvent in the sprayer tank (oil- or water-based), (ii) flow rate, and (iii) droplet size and volume.
    - For realistic and reproducible results, formulated EO products should ideally be diluted in water and applied using standardized spray systems (e.g., Potter spray tower or Peet–Grady chamber).
    - Experiments should be conducted in open or ventilated chambers to minimize vapor accumulation and reduce unintended inhalation effects.
    - Following treatment, insects should be transferred to clean, open containers until data collection. This prevents confounding effects due to residual vapors or unintended fumigant activity.
    - Environmental parameters such as temperature and relative humidity should be recorded, as these can influence droplet evaporation, EO volatility, and insect behavior.
  - Contact Bioassays via Impregnated Surface (Indirect Contact)
    - The EO-treated surface and test insects should be maintained in open, ventilated containers to allow differentiation between toxicity resulting from cuticular contact and that caused by inhalation.
    - Since many EOs exhibit repellency, the exposure surface should not be fully saturated. Untreated areas should be provided to allow insects the choice to avoid treated surfaces, better simulating field behavior.
    - Exposure time and surface area should be adjusted to reflect realistic field scenarios, especially for crawling or resting insects.
    - Residue persistence on surfaces over time should be quantified to understand the decline of efficacy under natural conditions.
  - Fumigant Bioassays
    - Ensure that the target insect species is susceptible to fumigation under field conditions.
    - Open or partially ventilated containers are preferred in the laboratory to prevent overestimation of EO efficacy due to accumulation of vapors.
    - If the experimental chamber is too small to accommodate a positive control (e.g., a commercial fumigant), meaningful comparisons cannot be conducted, and results may overstate the effectiveness of the EO.
    - Airflow, temperature, and container volume should be considered as these factors strongly influence on vapor distribution and insect exposure.
    - Where feasible, cages or chambers that better mimic the microclimate and spatial complexity of the target habitat should be used.
3. **General recommendations across all bioassays**
  - Ensure that doses are expressed in units that allow meaningful comparison with other laboratory and field studies (e.g., mg a.i./m^2^ or mg a.i per unit area).
  - Appropriate positive controls using well-characterized insecticides should be included to allow meaningful benchmarking of EO activity.
  - All methodological parameters should be reported in detail, including EO composition, formulation type, solvent, and application technique, to enhance reproducibility and comparability.
  - When feasible, multiple exposure routes in laboratory testing should be included to reflect real-world conditions, while clearly distinguishing between contact and inhalation effects.

By following these recommendations, laboratory assays can produce more realistic and transferable data, supporting the development of EO-based insecticides that are effective under field conditions.

## 5. Conclusions

The selection of the most appropriate application technique for real-world insect control depends on several factors, such as the type of insecticide, its main route of entry, formulation characteristics, and the biology, behavior, and feeding ecology of the target pest. Each insect species should therefore be managed using application methods specifically tailored to that pest.

As emphasized throughout this review, laboratory evaluations of EOs must use methods that realistically simulate field conditions. Inappropriate selection of bioassays can significantly overestimate of EO efficacy, producing results with limited practical use. Meaningful toxicity data can only be obtained when the route, application method, and solvent system accurately simulate the intended field applications in insect pest control.

Ensuring that laboratory protocols meet regulatory efficacy standards is crucial for advancing EO-based insecticides toward commercial biopesticide development. Additionally, field trials are essential, as they provide the most reliable assessment of product performance under real-world environmental, biological, and operational conditions.

Our work demonstrates that methodological choices in laboratory assays, such as topical application, exposure to treated surface or fumigation in enclosed spaces, can produce significant overestimations of efficacy of EOs. This laboratory–field efficacy gap constitutes a critical initial barrier that must be overcome before confronting the well-established commercialization challenges articulated by Isman [100]. There are three main barriers that can be re-examined in light of our analysis. First, regarding the sustainability of the botanical resource, the high efficacy from unrealistic laboratory tests may lead to field applications without a realistic assessment of the effective dose per hectare. If field trials based on overestimated laboratory data require impractical quantities of active ingredient, the sustainability and economic viability are immediately compromised. Realistic, field-simulative bioassays provide a more accurate early warning of this limitation. Second, the standardization of chemically complex extracts must extend beyond chemical composition to include biological standardization through methodologically sound, repeatable bioassays that reflect a specific mode of application. A standardized EO tested using an inappropriate bioassay provides little practical value. Therefore, developing commercially viable products requires the parallel standardization of both the input material and the efficacy testing protocol against defined field scenarios. Third, for regulatory approval processes, agencies such as the Environmental Protection Agency (EPA) or the European Commission (EU) require efficacy data generated under conditions that simulate practical use. Studies employing inappropriate solvents, exaggerated doses, or exposure routes not relevant to the proposed product label are likely to be dismissed. Consequently, bridging the methodological gap identified in this review is not merely an academic concern but a prerequisite for generating the valid, transferable data packages required for regulatory submission. Addressing the methodological flaws contributes to lowering the regulatory barrier.

In summary, although the challenges of sourcing, standardization, and regulation are formidable, their resolution ultimately depends on reliable, field-predictive efficacy data from the outset. By adopting the more rigorous, application-aware laboratory practices advocated in this review, researchers can generate data that not only more accurately reflect the true potential of EO-based insecticides but more effectively support their progression toward sustainable, standardized, and regulatable commercial products.

## Figures and Tables

**Table 1:** Comparison of primary insecticide application techniques used in field conditions, with implications for laboratory bioassay design.

Application Technique	Typical Volume, Droplet Size (VMD) * and Formulations **	Primary Target Scenario	Key Laboratory Bioassay to Simulate	Critical Parameters to Replicate in Lab
High Volume (HV)/Residual Spraying	>400 L/ha 200–500 µm (e.g., suspension concentrate (SC), microencapsulated (ME), wettable powder (WP)	Crawling insects on surfaces; thorough wetting of foliage/structures for residual action.	Contact bioassay via topical application (spray) or impregnated surface (for residual effect).	Dose expressed as mass a.i. * per unit area (e.g., mg/m^2^); use of water-based dilutions; controlled deposition on representative surfaces (e.g., filter paper, leaf disks).
Ultra-Low Volume (ULV)/Space Spraying	<5 L/ha 0.1–50 µm (optimal for flying insects) (ULV formulation or emulsifiable concentrate (EC))	Flying insects (e.g., mosquitoes, flies); aerosol droplets remain airborne.	Contact bioassay via topical application with precise, very small droplets.	Droplet size is critical; requires equipment like Peet–Grady chamber; dose often expressed as volume a.i per unit area; rapid exposition and assessment before droplets settle.
Fumigation	Gas concentration over time (e.g., tablets, pellets, smoke-generating, liquid).	Insects in enclosed, gas-tight spaces (e.g., stored grain, soil under tarps).	Fumigant bioassay in sealed containers.	Vapor concentration and exposure time; requires gastight chambers; relevant only for pests controlled this way in practice.
Dusting	Powder formulation for direct use (e.g., dust or dustable powders (D)	Similarly to HV spraying but with dry particulates; often for specific contexts (e.g., horticulture, animal housing).	Impregnated surface or direct dust application.	Particle size distribution; evenness of application; difficult to replicate standardly, often best evaluated in semi-field tests.

* VMD: Volume Median Diameter; a.i.: active ingredient. ** Formulations: It refers to the recommended formulations for that technique.

## Data Availability

No new data were created or analyzed in this study. Data sharing is not applicable to this article.
